# Perineurioma Diagnosed After Endoscopic Treatment of Suspected Gastric Adenocarcinoma of the Fundic Gland

**DOI:** 10.14309/crj.0000000000001110

**Published:** 2023-08-14

**Authors:** Ryohsuke Yokosuka, Takashi Ikeya, Hiroshi Yamato, Naoki Kanomata, Katsuyuki Fukuda

**Affiliations:** 1 Department of Gastroenterology, St. Luke's International Hospital, Tokyo, Japan; 2 Department of Pathology, St. Luke's International Hospital, Tokyo, Japan

**Keywords:** biopsy, endoscopic submucosal dissection, nerve sheath neoplasms

## Abstract

Perineurioma is a relatively rare tumor with an occasionally difficult differential diagnosis. A 63-year-old woman underwent esophagogastroduodenoscopy, which revealed a 15 mm, slightly faded, flat, and elevated lesion in the gastric body. Biopsy revealed a bundle-like proliferation of spindle-shaped cells; however, the diagnosis was unconfirmed. Endoscopic submucosal dissection was performed for diagnosis and treatment. Histopathological examination of the lesion revealed cell proliferation with short spindle-shaped and oval nuclei and little atypia in the lamina propria. Immunohistochemical examination indicated a perineurioma. Thus, when spindle-shaped cells are found on biopsy, it is necessary to consider the possibility of perineurioma.

## INTRODUCTION

Perineurioma is a rare soft-tissue tumor with extremely rare occurrence in the gastrointestinal tract. There are limited reports on perineuriomas in the stomach, suggesting that they are not well-recognized. In some cases, perineurioma is not diagnosed by biopsy and differentiation from other malignant tumors, such as gastric adenocarcinoma of the fundic gland (GAFG), is challenging. We report a case in which endoscopic submucosal dissection (ESD) was performed for the diagnostic treatment of a fading, flat, and elevated lesion. Histopathological examination revealed a perineurioma.

## CASE REPORT

A 63-year-old woman underwent screening esophagogastroduodenoscopy in 2021, and a 15 mm, slightly faded, flat, and elevated lesion with an irregular glandular duct structure was detected in the greater curvature of the gastric body. Because the background mucosa showed no atrophy and the patient was uninfected with *Helicobacter pylori*, a biopsy was performed owing to the suspicion of GAFG. Histopathological examination revealed a bundle-like proliferation of spindle-shaped cells; however, no findings revealed the presence of a gastrointestinal stromal tumor (GIST) or other neoplastic lesions on gross appearance or immunohistochemical staining. Because of the small size, it was difficult to determine whether the lesion was neoplastic or reactive; thus, GAFG was suggested as a possible differential diagnosis. Therefore, the patient was referred to us for further examination and treatment.

**Figure 1.**
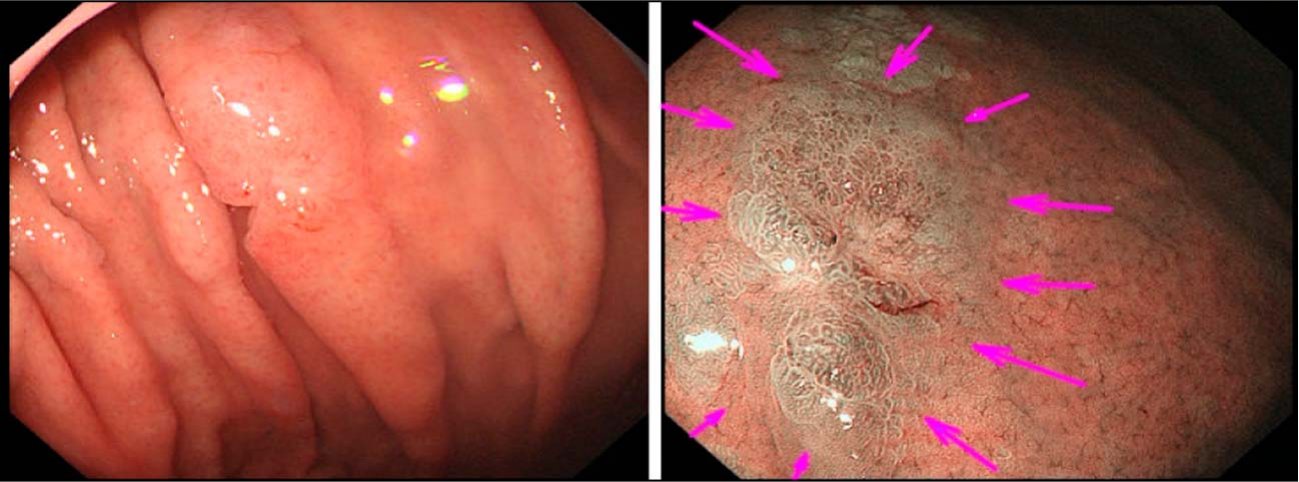
Esophagogastroduodenoscopy at the initial presentation. A, White-light endoscopy reveals a slightly faded, 15 mm, flat, and elevated lesion on the greater curvature of the gastric body. B, Narrow-band imaging-magnified endoscopy reveals an unclear demarcation line and no structural irregularities other than biopsy scars.

A second esophagogastroduodenoscopy revealed similar results as obtained on previous screening. The center of the lesion was a depressed epithelium, as observed after biopsy (Figure 1). Narrow-band imaging-magnified endoscopy revealed no obvious structural irregularities other than a biopsy scar. The vasculature was partially dilated, and the demarcation of the lesion was unclear (Figure 1).

**Figure 2.**
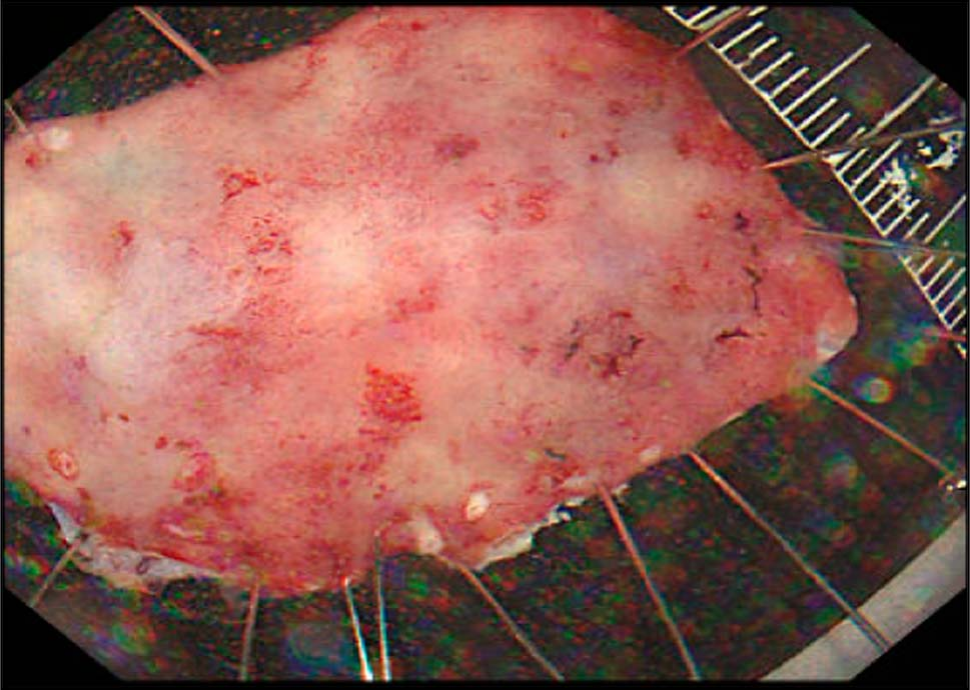
Gastric endoscopic submucosal dissection resection specimen. The resected specimen measured 30 × 20 mm, and the lesion measured 25 × 15 mm.

The possibility of GAFG could not be ruled out based on the endoscopic findings. Therefore, ESD was performed for diagnostic treatment, and the lesion was successfully resected en bloc without any adverse events (Figure 2).

**Figure 3.**
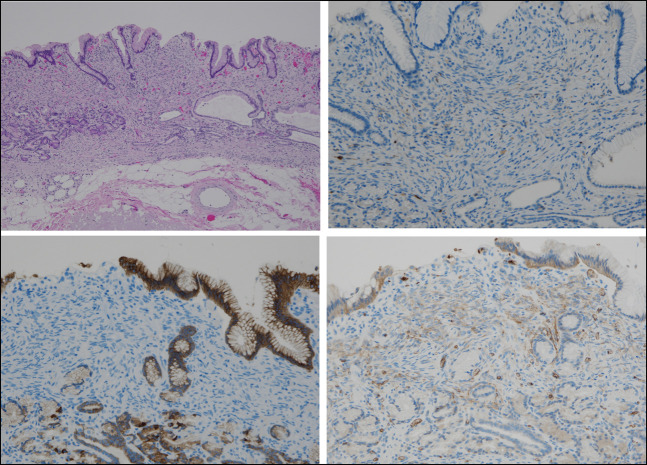
Gastric endoscopic submucosal dissection resection specimen. A, Hematoxylin and eosin staining (4×): Cell proliferation with short spindle-shaped/oval nuclei with little atypia is observed in the lamina propria. B, S100 immunostaining (10×). C, Epithelial membrane antigen immunostaining (10×). D, GLUT1 immunostaining (10×). The findings were consistent with those of a perineurioma.

Histopathological examination revealed cell proliferation with short spindle-shaped/oval nuclei and little atypia in the lamina propria. No evidence of necrosis or mitosis was observed (Figure 3). Immunohistochemical staining expressed GLUT1(+), CD34(+), epithelial membrane antigen (EMA) (−), S100(−), CD117(−), DOG1(−), desmin(−), AE1/3(−), and Ki67 < 1%. The size of the tumor was approximately 4 × 4 mm, without any malignant feature (Figure 3). Based on these findings, the patient was diagnosed with a perineurioma.

**Figure 4.**
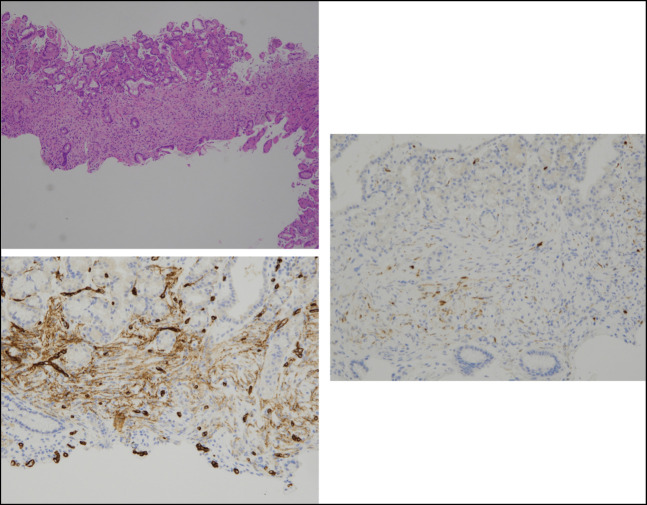
Previous biopsy specimen. A, Hematoxylin and eosin staining (4×): The background is basal gastric mucosa, and short spindle-shaped cell proliferation with little atypia is observed in the lamina propria. B, CD34 immunostaining (10×). C, S100 immunostaining (10×). The findings were consistent with those of a perineurioma.

After diagnosis, the pathological specimen from the previous biopsy was retrospectively re-examined. Cell proliferation with short spindle-shaped/oval nuclei and minimal atypia was observed within the lamina propria with no evidence of necrosis or mitosis. Immunohistochemical staining expressed CD34(+), S100(−), CD117(−), desmin(−), αSMA(−), and Ki67 < 1%. These findings were similar to those observed in gastric ESD specimens and were consistent with those of a perineurioma (Figure 4).

## DISCUSSION

Perineuriomas are peripheral nerve sheath tumors derived from perineural cells. They are rare, accounting for <1% of all soft-tissue tumors. Perineuriomas of the gastrointestinal tract were first described by Eslami-Varzaneh et al in 2004 and were previously called benign fibroblastic polyps.^1^ Perineuriomas of the gastrointestinal tract are extremely rare, and most commonly occur in the sigmoid colon. There have been no reports of death due to metastasis or recurrence because of the benign nature of the tumor. However, it is important to differentiate it from other neoplastic lesions to prevent unnecessary invasive treatment.^2^

Although endoscopic findings have not been mentioned in many previous reports, perineuriomas are reported to exhibit a yellow or faded color, solitary aphthous polyp, or submucosal tumor-like morphology.^3–5^ The surface is smooth, and the pit pattern is sparse or of type I. In a report, narrow-band imaging-magnified endoscopy revealed a regular vascular pattern and slightly thick dendritic vessels extending radially.^6^ The grossly visualized morphology of the lesion was diverse. However, the findings from this case are inconsistent with those of some studies that reported GIST, carcinoid, GAFG, and metastatic gastric tumor as the differential diagnoses.

A comparative study of perineurioma cases in the gastrointestinal tract has been reported in Japan, with only 4 reported cases of perineurioma in the stomach.^7^ Two cases were reported in the gastric body, 1 in the fornix and 1 in the antrum. Gross findings were polyp-like in one of the 4 cases and submucosal tumor-like in the other 3 cases.

Similarly, in this case, the lesion was located in the gastric body, and the endoscopic findings were consistent with those of previous reports. Specific immunohistochemical markers for neurofibromatous cells, such as EMA, GLUT1, and claudin-1, have been reported to be effective for the diagnosis of perineurioma; however, EMA staining is weak and occasionally negative.^2,8^ Other tumor-like lesions composed of spindle-shaped cells that should be differentiated include GIST and schwannoma. In the case of GIST, differentiation is made based on the positivity of CD117 and DOG1, whereas in schwannoma, differentiation is based on the positivity of S-100. In this case, EMA and S-100 were negative, but GLUT1 was positive, leading to the diagnosis of perineurioma. In addition, minimal cytologic atypia and negativity for AE1/3 in this case were useful findings to exclude the diagnosis of adenocarcinoma.

GAFG has recently attracted attention as gastric cancer unrelated to *H. pylori* infection, and its endoscopic findings include a faded color tone, submucosal tumor-like elevated lesions, dendritic vascular dilation, and background mucosa of the gastric fundic gland.^9^ Histopathological examination revealed a cellular image similar to that of a normal fundic gland. Therefore, a definitive diagnosis by biopsy is sometimes difficult.^10^

In this study, after the diagnosis of the perineurioma, we obtained histopathological specimens from the previous hospital, retrospectively examined the findings, and confirmed them to be consistent with those of a perineurioma. In recent years, ESD has been increasingly applied for the diagnosis and treatment of submucosal tumor-like tumors and *H. pylori*-negative gastric tumors in the stomach, with a suspicion of GAFG.

In conclusion, we believe that it is necessary to consider a perineurioma in advance when spindle-shaped cells are observed on biopsy. By considering a perineurioma at the time of biopsy, it is possible to differentiate perineurioma from malignant tumors, such as GAFG or GIST, and avoid unnecessary invasive treatment.

## DISCLOSURES

Author contributions: All authors participated in writing and editing the manuscript. R. Yokosuka is the article guarantor.

Financial disclosure: None to report.

Previous presentation: This case report was presented at the 370th Congress of the Japanese Society of Gastroenterology; July 16, 2022; Kanto Branch, Japan.

Informed consent was obtained for this case report.

## References

[1] Eslami-Varzaneh F Washington K Robert ME . Benign fibroblastic polyps of the colon: A histologic, immunohistochemical, and ultrastructural study. Am J Surg Pathol. 2004;28(3):374–8. 15104300 10.1097/00000478-200403000-00010

[2] Hawes SN Shi J. Gastric perineurioma: Clinicopathological characteristics. Pathology. 2017;49(4):444–7. 28438389 10.1016/j.pathol.2016.12.349

[3] Muguruma N Okamura S Imoto Y . Perineurioma: An uncommon lesion in the gastrointestinal tract. Endoscopy. 2012;44(Suppl 2):E182–3. 22622734 10.1055/s-0031-1291748

[4] Agaimy A Wuensch PH. Perineurioma of the stomach. A rare spindle cell neoplasm that should be distinguished from gastrointestinal stromal tumor. Pathol Res Pract. 2005;201(6):463–7. 16136753 10.1016/j.prp.2005.05.012

[5] Chetty R. Myxoid perineurioma presenting as a gastric polyp. Ann Diagn Pathol. 2010;14(2):125–8. 20227017 10.1016/j.anndiagpath.2009.06.002

[6] Fujino Y Muguruma N Kitamura S . Perineurioma in the sigmoid colon diagnosed and treated by endoscopic resection. Clin J Gastroenterol. 2014;7(5):392–6. 26184017 10.1007/s12328-014-0519-x

[7] Matsui S Hiroshi K Rie T . Gastric perineurioma with subepithelial tumor-like appearance, report of a case. Stomach Intest. 2017;52(8):1098–106.

[8] Hornick JL Fletcher CDM. Intestinal perineuriomas: Clinicopathologic definition of a new anatomic subset in a series of 10 cases. Am J Surg Pathol. 2005;29(7):859–65. 15958849 10.1097/01.pas.0000154130.87219.2c

[9] Ueyama H Matsumoto K Nagahara A Hayashi T Yao T Watanabe S. Gastric adenocarcinoma of the fundic gland type (chief cell predominant type). Endoscopy. 2014;46(2):153–7. 24338239 10.1055/s-0033-1359042

[10] Singhi AD Lazenby AJ Montgomery EA. Gastric adenocarcinoma with chief cell differentiation: A proposal for reclassification as oxyntic gland polyp/adenoma. Am J Surg Pathol. 2012;36(7):1030–5. 22472957 10.1097/PAS.0b013e31825033e7

